# Human inborn errors of long‐chain fatty acid oxidation show impaired inflammatory responses to TLR4‐ligand LPS

**DOI:** 10.1096/fba.2024-00060

**Published:** 2024-08-19

**Authors:** Signe Mosegaard, Krishna S. Twayana, Simone W. Denis, Jeffrey Kroon, Bauke V. Schomakers, Michel van Weeghel, Riekelt H. Houtkooper, Rikke K. J. Olsen, Christian K. Holm

**Affiliations:** ^1^ Research Unit for Molecular Medicine, Department of Clinical Medicine Aarhus University and Aarhus University Hospital Aarhus Denmark; ^2^ Laboratory Genetic Metabolic Diseases, Amsterdam UMC University of Amsterdam Amsterdam The Netherlands; ^3^ Amsterdam Gastroenterology, Endocrinology, and Metabolism Amsterdam The Netherlands; ^4^ Amsterdam Cardiovascular Sciences Amsterdam The Netherlands; ^5^ Department of Biomedicine, Aarhus Research Center for Innate Immunology Aarhus University Aarhus Denmark; ^6^ Laboratory of Angiogenesis and Vascular Metabolism VIB‐KU Leuven Center for Cancer Biology, VIB Leuven Belgium; ^7^ Laboratory of Angiogenesis and Vascular Metabolism, Department of Oncology KU Leuven and Leuven Cancer Institute (LKI) Leuven Belgium; ^8^ Core Facility Metabolomics Amsterdam University Medical Centers, University of Amsterdam Amsterdam The Netherlands; ^9^ Emma Center for Personalized Medicine Amsterdam UMC Amsterdam The Netherlands

**Keywords:** immunometabolism, lipopolyssacharide, long‐chain fatty acid oxidation disorders, toll‐like receptor 4

## Abstract

Stimulation of mammalian cells with inflammatory inducers such as lipopolysaccharide (LPS) leads to alterations in activity of central cellular metabolic pathways. Interestingly, these metabolic changes seem to be important for subsequent release of pro‐inflammatory cytokines. This has become particularly clear for enzymes of tricarboxylic acid (TCA) cycle such as succinate dehydrogenase (*SDH*). LPS leads to inhibition of SDH activity and accumulation of succinate to enhance the LPS‐induced formation of IL‐1β. If enzymes involved in beta‐oxidation of fatty acids are important for sufficient responses to LPS is currently not clear. Using cells from various patients with inborn long‐chain fatty acid oxidation disorders (lcFAOD), we report that disease‐causing deleterious variants of Electron Transfer Flavoprotein Dehydrogenase (*ETFDH*) and of Very Long Chain Acyl‐CoA Dehydrogenase (*ACADVL*), both cause insufficient inflammatory responses to stimulation with LPS. The insufficiencies included reduced TLR4 expression levels, impaired TLR4 signaling, and reduced or absent induction of pro‐inflammatory cytokines such as IL‐6. The insufficient responses to LPS were reproduced in cells from healthy controls by targeted loss‐of‐function of either *ETFDH* or *ACADVL,* supporting that the deleterious *ETFDH* and *ACADVL* variants cause the attenuated responses to LPS. *ETFDH* and *ACADVL* encode two distinct enzymes both involved in fatty acid beta‐oxidation, and patients with these deficiencies cannot sufficiently metabolize long‐chain fatty acids. We report that genes important for beta‐oxidation of long‐chain fatty acids are also important for inflammatory responses to an acute immunogen trigger like LPS, which may have important implications for understanding infection and other metabolic stress induced disease pathology in lcFAODs.

## Graphical abstract

We report that inactivating gene variants of Electron Transfer Flavoprotein Dehydrogenase (*ETFDH*) or Very Long Chain Acyl‐CoA Dehydrogenase (*ACADVL*) caused insufficient responses to Lipopolysaccharide (LPS) which was also mimicked in cells from normal healthy controls under targeted loss‐of‐function of same genes. It included impaired TLR4 signaling and reduced or absent induction of pro‐inflammatory cytokines like IL‐6. This links inborn errors in long‐chain fatty acid oxidation (lcFAOD) to insufficient responses to LPS, which may have important implications for understanding infection‐induced disease pathology in lcFAODs.

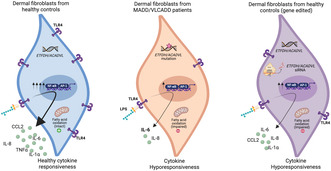

## 1 INTRODUCTION

Metabolic pathways are crucial for the proper functioning of the cell and are involved in various processes such as energy production, biosynthesis, and degradation of molecules.^1^ It has now become clear that these metabolic pathways also play critical roles in the regulation of immune responses.^2^, ^3^, ^4^ For example, pharmacological inhibition of glycolysis also inhibits lipopolysaccharide (LPS)‐induced formation of the pro‐inflammatory cytokine IL‐1.^5^ Intriguinly, the dependency on glycolysis for the response to LPS is based on stabilization of Hypoxia‐inducible Factor 1‐alpha (HiF1α) by an LPS‐induced increment in succinate levels.^5^ Later, the increment in succinate was demonstrated to be dependent on another metabolite itaconate derived from aconitate.^6^ These discoveries were among the first to demonstrate that enzymes and metabolites of the tricarboxylic acid (TCA) cycle impacted inflammatory responses to LPS. Fatty acid oxidation (FAO) also intersects with inflammatory responses and has been associated with development of anti‐inflammatory phenotypes of macrophages. Indeed, induction of classical differentiation of macrophages through stimulation with LPS or Interferon gamma (IFNγ) induce an increase in glycolytic flux. By contrast, alternative activation induced by IL‐4 seemed to promote an increased reliance on FAO.^7^, ^8^ In line, forced increases in FAO in adipocytes seems to limit the pro‐inflammatory phenotype induced by treatment with palmitate.^9^ This link has recently been strengthened as stimulation of murine macrophages with LPS is now demonstrated to lead to an increase in FAO that involves stabilization of the mitochondrial fatty acid transporter carnitine palmitoyl transferease 1a (CPT1a).^10^ If FAO or central FAO enzymes are important for the pro‐inflammatory release of cytokines in the response to stimuli like LPS is not clear. Furthermore, observations of such connection in human disease have not yet been made.

Disorders of long‐chain fatty acid oxidation (lcFAOD) were first described in the 1970's, and since, inherited defects have been described for most of the enzymes, transporters, and other facilitating proteins involved in FAO.^11^, ^12^ lcFAODs are recessively inherited and lead to impaired energy production and various clinical symptoms including cardiac‐ and skeletal muscle myopathies. The most common lcFAOD is VLCAD deficiency (VLCADD), which is caused by genetic variants in *ACADVL*, and has a worldwide prevalence of 1:31.500–1:94.569.^13^ VLCADD can present with mild or severe disease, or even remain asymptomatic for years. A less frequent lcFAOD is multiple acyl‐CoA dehydrogenation deficiency (MADD or glutaric aciduria type II (GAII)). Similar to patients with VLCADD, patients with MADD vary in their clinical presentation. Mildly affected patients present similarly to VLCADD with mainly muscle‐related phenotypes such as exercise intolerance, weakness and pain. Severely affected MADD patients present with a multisystemic and often lethal disease. Most patients with MADD harbor genetic variants in genes encoding the electron transfer flavoprotein (ETF) or ETF‐ubiquinone oxidoreductase (ETF‐QO).^14^

It is known from clinical practice that infections are a disease trigger in lcFAODs,^15^, ^16^, ^17^ and that inflammation and infections can cause metabolic decompensation, rhabdomyolysis and even trigger multi‐organ failure and death,^14^, ^18^, ^19^, ^20^ but the mechanisms are yet unknown. In humans, only a few studies describe immune responses in lcFAODs. Those studies show an inflammatory dysregulation both during symptomatic and non‐symptomatic periods.^17^, ^21^, ^22^

Using samples from two MADD and six VLCADD patients, we here report that patient cells with inactivating gene variants in *ETFDH* or *ACADVL* display highly impaired responses to stimulation with LPS. This included reduced expression of TLR4, impaired TLR4 signaling, and reduced or absent induction of cytokines such as IL‐6. Our study thereby delivers genetic evidence using patient‐derived cells, that enzymes important for beta‐oxidation of long‐chain fatty acids are also critical for effective responses to stimulation with LPS. Since inflammatory factors like IL‐6 may play a protective role by regulating metabolic function and survival/regeneration of tissue upon stress exposure, our findings may have important implications for understanding infection‐induced disease pathology in lcFAODs.

## 2 MATERIALS AND METHODS

### 2.1 Patient and control material

Human primary fibroblasts were derived from patients previously diagnosed with severe or mild VLCADD or severe MADD, based on clinical, biochemical, and genetic data (Table S1). Control dermal fibroblasts were obtained from healthy individuals. All the cells were de‐identified, and the inclusion of these samples in this study is according to the Dutch and Danish ethical committee regulations.

### 2.2 Cell culture and reagents

Dermal fibroblasts from MADD (P1 and P2) and healthy controls (C1 and C2) were cultured in DMEM (Merk, D6429) supplemented with 10% heat inactivated fetal bovine serum (Merk, F9665), 200 IU/mL − 1 penicillin, 100 μg/mL − 1 streptomycin and 600 μg/mL − 1 glutamine, hereafter termed complete DMEM. Similarly, dermal fibroblasts from VLCADD patients (severe; P5, P6, P7, P8, and mild; P3 and P4) and controls (C3, C4, C5 and C6) were cultured until confluent with Ham's F‐10 nutrient mixture (Gibco) supplemented with L‐glutamine (Bio‐Whittaker), 10% fetal bovine serum (FBS) (Bio‐Whittaker), 100 U/mL penicillin, 100 μg/mL streptomycin (Life Technologies), and 250 ng/mL Fungizone (Life Technologies) in a humidified atmosphere with 5% CO_2_ at 37°C. When confluent, cells were seeded in biological duplicates or triplicates in 6 wells (200,000 cells/well) or 12 wells (100,000 cells/well) with equal confluency for 24 h. Medium was then replaced with Ham's F‐10 nutrient mixture, 1% FBS, as described above, with 0 ng/mL or 400 ng/mL lipopolysaccharide (LPS) (Invivogen (tlrl‐b5lps)), or 2 μg/mL poly(I:C) (Invivogen (trl‐pic‐5)) or for VLCADD (Serotype EH100 (RA) (TLRGRADE®) (Ready‐to‐Use), Alexis Biochemicals). Cells were harvested using trypsin, and washed twice with PBS, before cell pellets were frozen at −80°C. LPS concentration and duration of incubation were chosen based on published studies and several test experiments.^23^, ^24^, ^25^ Cells were regularly tested for mycoplasma contamination by sequencing from GATC Biotech (Germany).

### 2.3 Long‐chain fatty acid oxidation flux assay

FAO flux in MADD (P1, P2) and control cells (C1, C2) was determined by measuring radiolabelled H_2_O from [9,10‐^3^H(N)]‐ palmitic acid. FAO flux was assayed for controls, *ETFDH* mutant cells, and *ETFDH* silenced control fibroblasts. 100 μM Etomoxir (ETO), a small molecule inhibitor of CPTI, was used as positive control for FAO reduction. Cells seeded in 24 well plates (50,000 cells/well) were either transfected with siETFDH for 72 h, or pretreated with 100 μM ETO for 12 h, or directly proceeded for FAO measurement in case of controls and *ETFDH* mutant cells. Briefly, cells were incubated for 6 h with complete DMEM containing radiolabelled [9,10‐^3^H] palmitic acid. Supernatant was then used to determine radioactivity by liquid scintillation counting. Measurement was done at 37°C and FAO flux was expressed in nmol palmitate/h/mg protein. VLCADD (severe; P5, P6, P7, P8, and mild; P3 and P4) and control (C4, C5, and C6) dermal fibroblasts were seeded in 48‐well plates, and the oxidation fluxes were performed in duplicates and measured at 37°C by the production of ^3^H_2_O from [9,10–^3^H(N)]‐oleic acid as previously described.^26^

### 2.4 Enzyme‐linked immunoassay (ELISA)

Cytokines in the patient and control dermal fibroblast culture supernatants were measured using enzyme‐linked immunosorbent assay (ELISA) for quantitative detection of IL‐1β (R&D Systems Cat. No. DY201), tumor necrosis factor TNF‐α (BioLegend® Cat. No. 430204), IL‐6 (BioLegend® Cat. No. 430504 and R&D Systems Cat. No. DY206), IL‐8 (Thermo Scientific 88–8086), IL‐10 (BioLegend® Cat. No. 430604 and R&D Systems Cat. No. DY217B), and CXCL10 (R&D Systems Cat. No. DY266), all according to the manufacturer's instructions. Briefly, the plates coated with capture antibody overnight at room temperature (RT) were washed three times (PBS+0.05% Tween) followed by samples and standards addition to the wells and overnight incubation at 4°C. The wells were then washed and incubated with detection antibody at RT for 2h. Wells were washed again and conjugated with streptavidin HRP (R&D, 893975) at RT for 20 min, in the dark. After final washing, tetramethylbenzidine (TMB) substrate (Promega, 67431) was added till sufficient enzyme‐substrate reaction was visualized, which was then terminated by adding stop solution ((2N) sulfuric acid, VWR chemicals) and measured at 450 nm (570 nm for wavelength correction) using Synergy HTX multimode reader.

### 2.5 RNA analysis and QPCR analysis

In MADD and control fibroblasts, RNA from dermal fibroblasts cell pellets were extracted using High Pure RNA Isolation Kit (Roche) using manufacturer's instructions. RNA quantification and quality assessment was done using Nanodrop spectrometry (Thermo Fisher Scientific). Gene expression was determined by real‐time quantitative PCR (Taqman Gene Expression Assay, Gene Assay IDs in Table S6), using TaqMan detection systems (Applied Biosciences). mRNA levels were determined using Taqman RNA‐to‐Ct 1‐step Kit (Applied Biosystems) following manufacturer's guidelines.

RNA from VLCADD and control fibroblast cell pellets were isolated by using TRI Reagent® (Sigma Aldrich) according to the manufacturer's instructions. RNA was quantified using the NanoDrop 2000 spectrophotometer (Thermo Fisher Scientific). 1 μg of RNA was pre‐treated with gDNA Wipeout Buffer and reverse‐transcribed to cDNA according to the manufacturer's instructions using QuantiTect Reverse Transcription Kit (QIAGEN). Quantitative gene expression analysis was performed using the LightCycler®480 SYBR Green I Master (Roche) and measured using the LightCycler®480 Instrument II (Roche). Relative expression was calculated by LinRegPCR^27^ and the N0 values were normalized to the geometric mean of reference genes beta‐actin, GAPDH and 36B4. TaqMan® Gene Expression Assays IDs used for this study were *IL‐6* (Hs00174131_m1), *CCL2 (*Hs00234140_m1), *hTNF‐α* (Hs00230464_m1), *IL‐1α* (Hs00899844_m1), *CXCL10 (*Hs00171042_m1), and *TLR4 (*Hs04188203_s1).

### 2.6 Western blot

Cells lysate was collected in 100 μL of ice‐cold Pierce RIPA lysis buffer (Thermo Scientific) supplemented with 10 mM NaF, 1x complete protease cocktail inhibitor (Roche) and 5 IU mL − 1 benzonaze (Sigma), respectively. Protein concentration was determined using a BCA protein assay kit (Thermo Scientific). Protein denaturation before separation was done by treating the cell lysates for 3 min at 95°C in presence of 1x XT Sample Buffer (BioRad) and 1x XT reducing agent (BioRad). 15 to 30 μg of protein sample was processed for separation through SDS PAGE on 4%–20% Criterion TGX precast gradient gels (BioRad). Gel run consisted of 15 min run at 70 V followed by 45 min run at 100 V. A dry protein transfer onto PVDF membrane (BioRad) was then performed using a Trans‐Blot Turbo Transfer system for 7 min. In order to prevent nonspecific binding, membranes were blocked for 1 h in 5% skim‐milk (Sigma Aldrich) at room temperature in PBS supplemented with 0.05% Tween‐20 (PBST). Blocked membranes were cut according to desired protein molecular weight and incubated overnight at 4°C in specific primary antibodies prepared in PBST (diluted 1:1000 unless mentioned). The one used for this study were: anti‐TLR4 (sc‐293,072, Santa Cruz Biotechnology), anti‐IRAK1 (#4504, CST), anti‐P‐IRAK1(# PA5‐38633, Thermo Fischer Scientific), anti‐IκBα (#4814, CST), anti‐Phospho‐IκBα (#2859, CST), anti‐NF‐κB p65 (#8242, CST), anti‐Phospho NF‐κB p65 (#3033, CST), anti‐ETFDH (sc‐515,202, Santa Cruz Biotechnology), anti‐JunB (#3753, CST), anti‐cJun (#9165, CST), anti‐FRA1 (#5281, CST), anti Phospho FRA1 (#3880, CST), and anti‐Vinculin (#13901, CST). Membranes were then washed three times in PBST and incubated in secondary antibodies; peroxidase‐ conjugated F(ab)2 donkey anti‐rabbit IgG (H + L) (1:10,000) (Jackson ImmunoResearch) prepared in 1% PBST skim milk. It was then followed by three times membranes washing in PBST and exposure to either the SuperSignal West Pico PLUS chemiluminescent substrate or the SuperSignal West Femto maximum sensitivity substrate (ThermoScientific) and Images were captured using an ImageQuant LAS4000 mini‐Imager (GE Healthcare).

### 2.7 Short‐interfering RNA (siRNA)‐mediated knockdown

For short interfering RNA experiments, cells seeded in 12 well plate (100,000 cells per well) were transfected with 80 pmol of gene specific siRNA human *ETFDH* (sc‐89,048), human *JunB* (sc‐35,726), human *MTOR* (sc‐35,409) or control siRNA (sc‐37,007), diluted in antibiotic free DMEM, using Lipofectamine RNAi Max as per manufacturer's instructions for 72 h. LPS 400 ng/mL stimulation was given after siRNA incubation without removing media.

### 2.8 CRISPR/Cas9 knockout of *ETFDH* and activation of *JunB*

Control dermal fibroblasts (300,000 to 800,000 cells), resuspended in 20 μL Opti‐MEM were electroporated with 1.5 nmol *ETFDH* guide RNA and 1.2 μL Cas9 enzyme. Cells were then seeded in prewarmed DMEM and grown until confluency for 2 to 3 days. These once knocked out cells were again collected and electroporated with similar composition of guide RNA and Cas9. Double knocked out cells were then grown for 2–3 days and seeded for the experiment. Cells were similarly electroporated with 1.5 nmol *JunB* guide RNA for activation, grown until confluency for 2 days and treated with 0 ng/mL or 400 ng/mL LPS without media removal. The *ETFDH* gene knockout or *JunB* gene activation were examined through western blot. For knockout, *ETFDH* guide RNA (ETFDH_g1, SYNTHEGO) used had sequence of GACCAUCUUGUAGCACAUAG.

### 2.9 Metabolomics

Metabolomics analysis was performed as previously described by Molenaars et al.^28^ Dermal fibroblasts from both VLCADD patients, MADD patients and control individuals were seeded in triplicates in 35 mm cell culture dishes with a density of 150,000 cells/dish in Ham's F‐10 nutrient mixture (Gibco) supplemented with L‐glutamine (Bio‐Whittaker), 10% foetal bovine serum (FBS) (Bio‐Whittaker), 100 U/mL penicillin, 100 μg/mL streptomycin (Life Technologies), and 250 ng/mL Fungizone (Life Technologies) in a humidified atmosphere with 5% CO_2_ at 37°C. After 24 h medium was changed to Ham's F‐10 nutrient mixture, 1% FBS (as described above) with 0 ng/mL or 400 ng/mL LPS (Serotype EH100 (RA) (TLRGRADE®) (Ready‐to‐Use), Alexis Biochemicals) and incubated for 24 h (5% CO_2_ at 37°C). On ice, medium was removed and stored at −20°C before the following solvents were added to each well: 500 μL methanol, 425 μL Milli‐Q, and 75 μL internal standard solution (in house solution, the full list available in Supplementary Materials). Cells were scraped off the dishes and both cells and solvents were pipetted into a 2 mL Eppendorf Safe‐lock tube and stored at −80°C. Samples were prepared for analysis by adding 1 mL chloroform followed by 1.5 min of thorough mixing, and subsequent centrifugation at 20000×*g*, 4°C for 10 min. Top phase was transferred to a 1.5 mL Eppendorf tube and dried using a vacuum concentrator at 60°C. Pellets were reconstituted in 100 μL 3:2 methanol: Milli‐Q and samples were mixed thoroughly, before centrifugation at 20000×*g*, 4°C for 10 min. 85 μL of each sample were transferred to a glass vial and stored at −20°C until mass spectrometry analysis. Metabolites were analyzed using a Waters Acquity ultra‐high performance liquid chromatography system coupled to a Bruker Impact II™ Ultra‐High Resolution Qq‐Time‐Of‐Flight mass spectrometer. Samples were kept at 12°C during analysis and 5 μL of each sample was injected. Chromatographic separation was achieved using a Merck Millipore SeQuant ZIC‐cHILIC column (PEEK 100 × 2.1 mm, 3 μm particle size). Column temperature was held at 30°C. Mobile phase consisted of (A) 1:9 (v/v) acetonitrile: water and (B) 9:1 (v/v) acetonitrile: water, both containing 5 mmol/L ammonium acetate. Using a flow rate of 0.25 mL/min, the LC gradient consisted of: 100% B for 0–2 min, reach 54% B at 13.5 min, reach 0% B at 13.51 min, 0% B for 13.51–19 min, reach 100% B at 19.01 min, 100% B for 19.01–19.5 min. Equilibrate column by increasing flow rate to 0.4 mL/min at 100% B for 19.5–21 min. MS data were acquired using negative and positive ionization in full scan mode over the range of *m*/*z* 50–1200. Data were analyzed using Bruker TASQ software version 2021.1.2452. All reported metabolite intensities were normalized to total protein content in samples, determined using a Pierce™ BCA Protein Assay Kit, as well as to internal standards with comparable retention times and response in the MS. Metabolite identification was based on a combination of accurate mass, (relative) retention times and fragmentation spectra, compared to the analysis of a library of standards.^28^, ^29^ The internal standard solution content for this metabolomics investigations consisted of adenosine‐15 N5‐monophosphate (5 nmol), adenosine‐ 15 N5‐triphosphate (5 nmol), D4‐alanine (0.5 nmol), D7‐arginine (0.5 nmol), D3‐aspartic acid (0.5 nmol), D3‐carnitine (0.5 nmol), D4‐citric acid (0.5 nmol), 13C1‐citrulline (0.5 nmol), 13C6‐ fructose‐1,6‐diphosphate (1 nmol), guanosine‐15 N5‐monophosphate (5 nmol), guanosine‐ 15 N5‐triphosphate (5 nmol), 13C6‐glucose (10 nmol), 13C6‐glucose‐6‐phosphate (1 nmol), D3‐glutamic acid (0.5 nmol), D5‐glutamine (0.5 nmol), D5‐glutathione (1 nmol), 13C6‐ isoleucine (0.5 nmol), D3‐lactic acid (1 nmol), D3‐leucine (0.5 nmol), D4‐lysine (0.5 nmol), D3‐ methionine (0.5 nmol), D6‐ornithine (0.5 nmol), D5‐phenylalanine (0.5 nmol), D7‐proline (0.5 nmol), 13C3‐pyruvate (0.5 nmol), D3‐serine (0.5 nmol), D6‐succinic acid (0.5 nmol), D5‐ tryptophan (0.5 nmol), D4‐tyrosine (0.5 nmol), D8‐valine (0.5 nmol).

### 2.10 Statistical analyses

For all experiments, biological cell culturing duplicates or triplicates have been included, indicated for each individual experiment in the figure legends. For some experiments, indicated in figure legends, also technical replicates have been included in the statistical analysis. Statistical significance between treated and non‐treated individual cell lines are calculated using unpaired Student's *t*‐test. All statistical analyses have been performed using GraphPad Prism 9 (GraphPad Software, Inc). Not significant (not indicated): *p* ≥ 0.05, *p = 0.01 to 0.05, **p = 0.001 to 0.01, ***p < 0.001, and ****p < 0.0001.

## 3 RESULTS

### 3.1 Deleterious *ETFDH* gene variants of MADD patients cause impaired responses to LPS

**FIGURE 1:**
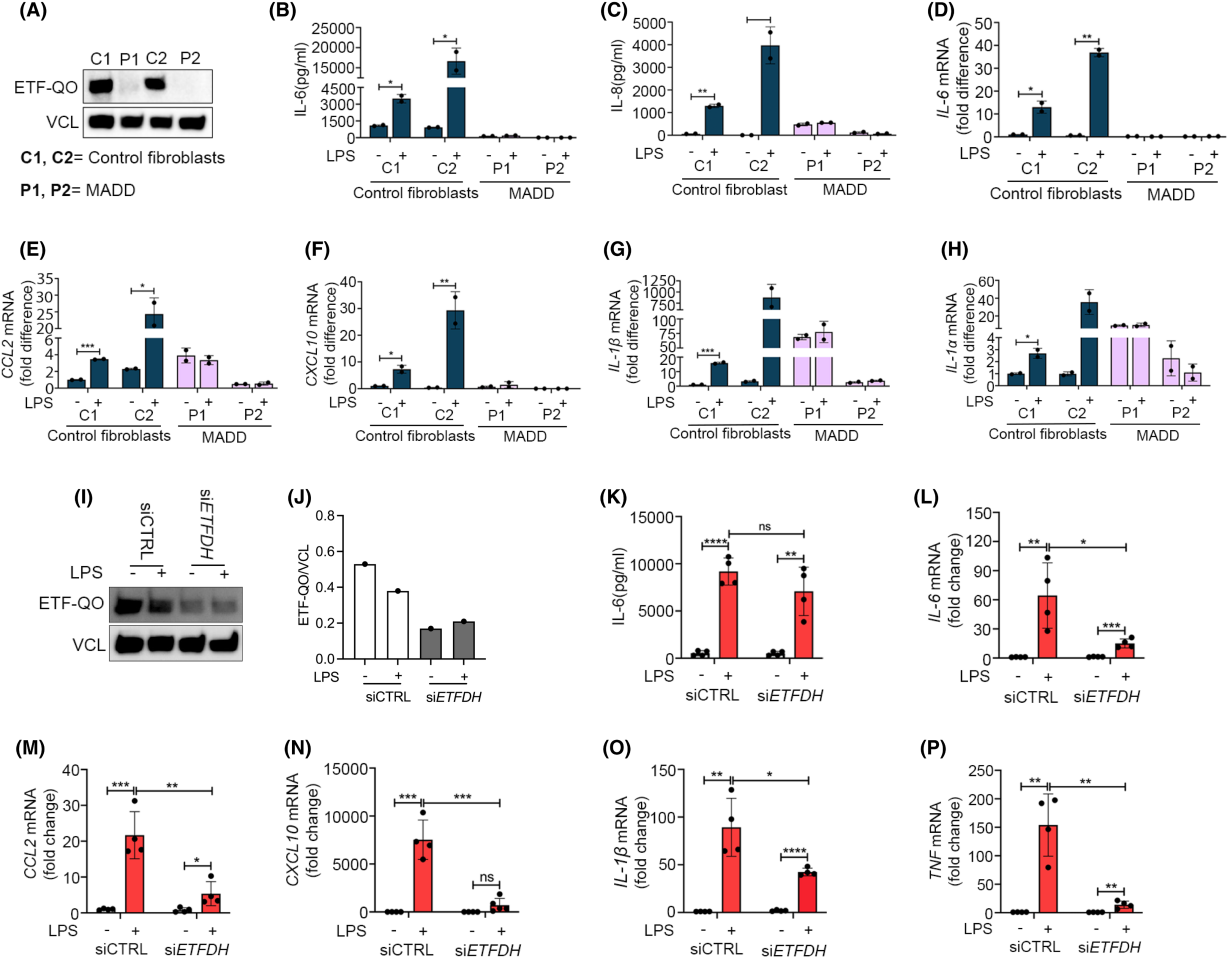
Inborn errors in *ETFDH* cause impaired responses to LPS. Primary dermal fibroblasts from healthy controls (C1‐2) and primary dermal fibroblasts derived from MADD patients (P1‐2) were (A) blotted for basal expression level of ETF‐QO. The cells were stimulated with LPS 400 ng/mL, and analyzed for (B, C) IL‐6, IL‐8 secretion at 24 h by ELISA or (D–H) cytokines mRNA (IL‐6, CCL2, CXCL10, IL‐1β, and IL‐1α) expression by RT‐QPCR at 6 h. Control dermal fibroblasts (C2) transfected with scrambled siRNA or *ETFDH* siRNA for 72 h were stimulated with LPS 400 ng/mL for (I–K) 24 h, blotted and quantified for ETF‐QO, and analyzed for IL‐6 secretion or (L–P) 6 h, and analyzed for cytokines mRNA (IL‐6, CCL2, CXCL10, IL‐1β, and TNF) expression by RT‐QPCR. Blots in figures (A and I) are representative of three independent experiments. Data in figures (B–D) and (K–P) are presented as mean ± SEM of four cell culture experiments, while data in figures (E–H) represent mean ± SEM of two cell culture experiments. *p < 0.05, **p < 0.01 ***p < 0.001, and ****p < 0.0001; compared to untreated control cells (unpaired *t* test). MADD, Multiple Acyl‐CoA Dehydrogenase Deficiency; ETFDH, Electron Transfer Flavoprotein Dehydrogenase.

We investigated the cytokine response to LPS using dermal fibroblasts derived from two patients each carrying deleterious variants of *ETFDH*, ecoding the ETF‐ubiquinone oxidoreductase (ETF‐QO) (Table S1).^30^, ^31^ The patient variants are essentially null‐variants as the *ETFDH*‐encoded product ETF‐QO is not expressed in the patient derived cells (Figure 1A). In short, patient‐derived fibroblasts (P1 + P2) and fibroblasts derived from healthy controls (C1 + C2) were stimulated with 400 ng/mL LPS. Cell culture medium was then collected to determine secretion of common pro‐inflammatory cytokines IL‐6 and IL‐8 by ELISA. Here, fibroblasts from healthy donors displayed significant LPS‐induced release of these cytokines. By contrast, the LPS‐induced cytokine release was markedly attenuated in fibroblasts derived from MADD patients (Figure 1B,C and Figure S1A). This also seemed to be the case when measuring cytokine induction at the mRNA level by qPCR for *IL‐6*, *IL‐8*, *CCL2*, *IL‐1β*, and *IL‐1α*, (Figure 1D–H). We also checked if the IL‐6 response is impaired similarly for another inflammatory stimuli—poly I:C; a well‐known TLR3 agonist. The overnight treatment of poly I:C 2 μg/mL significantly induced IL‐6 secretion in healthy controls (C1, C2). The induction was also observed in MADD patient derived fibroblasts P1, but not in P2. However, this induced IL‐6 level in P1 was significantly lower, compared to the induction in healthy control fibroblasts (Figure S1B). We then analyzed the basal mRNA levels of the TLR3 receptor. In line with IL‐6 secretion, the basal *TLR3* mRNA expression was significantly higher in healthy control fibroblasts (C1, C2) as compared to the patient fibroblasts, and with MADD patient P2 showing the lowest expression (Figure S1C).

Because the two included MADD patients carry genetically distinct deleterious variants of *ETFDH* (Table S1), it is highly plausible that the shared inability to respond to LPS originates from their deficiencies in *ETFDH*. Nevertheless, we wanted to explore further, if *ETFDH* deficiency in itself is sufficient to suppress cytokine responses to LPS thus linking the known genotype directly to the phenotype. For this purpose, we silenced *ETFDH* expression using siRNAs in fibroblasts C2 derived from healthy donors. Here, treatment with siRNA reduced the expression of ETF‐QO (Figure 1I,J), as well as induction of *IL‐6*, *CCL2*, *CXCL10*, *IL‐1β*, and *TNF* in response to stimulation with LPS as compared with cells treated with control siRNA (Figure 1K–P). These experiments support that the *ETFDH* deficiencies characterizing the MADD patients are sufficient to cause the insufficient response to LPS.

### 3.2 MADD patient cells have reduced TLR4 expression and signaling

**FIGURE 2:**
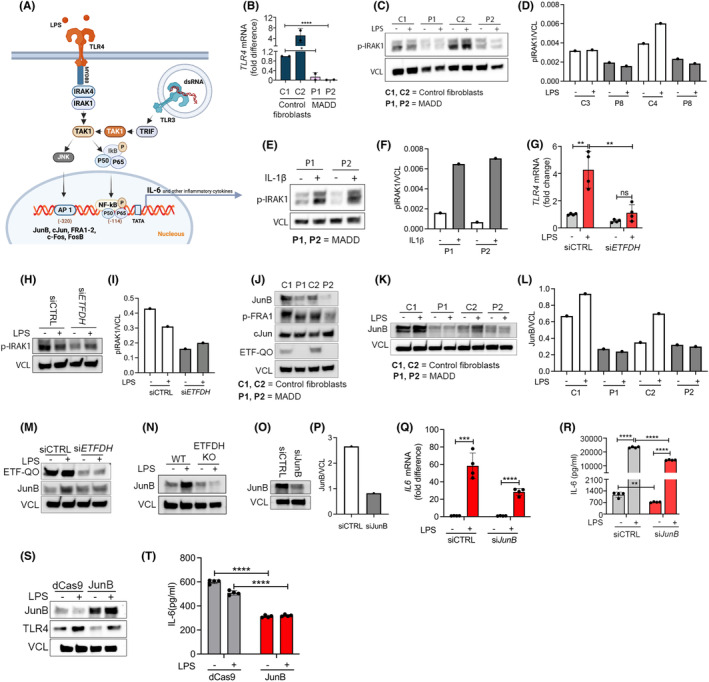
MADD patient cells have reduced TLR4 expression and signaling. (A) Selected proteins, signaling molecules and transcription factors involved in TLR4 and TLR3 signaling pathways. Primary dermal fibroblasts from healthy controls (C1‐2) and primary dermal fibroblasts derived from MADD patients (P1‐2) were (B) analyzed for basal TLR4 mRNA expression by RT‐QPCR, (C–F) stimulated with LPS 400 ng/mL or recombinant human IL‐1β 100 pg/mL for 15 min, blotted and quantified for phospho‐IRAK1. The cells were (J) also blotted for basal expression of AP‐1 transcription factors including JunB, phospho‐FRA1, and cJun, or (K, L) stimulated with LPS 400 ng/mL for 24 h and blotted for JunB. Primary dermal fibroblasts from healthy controls (C2) transfected with scrambled siRNA or *ETFDH* siRNA for 72 h were stimulated with LPS 400 ng/mL and (G) analyzed for TLR4 mRNA expression at 6 h, (H, I) blotted for phospho‐IRAK1 at 2 h, and (M) blotted for ETF‐QO and JunB at 24 h. (N) Control dermal fibroblasts (C2): Both wild type (WT) and those electroporated with ETFDH sgRNA for ETF‐QO knockout, were stimulated with LPS 400 ng/mL for 24 h and blotted for JunB. (O–R) Primary dermal fibroblasts from healthy control (C2) transfected with scrambled siRNA or JunB siRNA for 72 h and stimulated with LPS 400 ng/mL. Cells were analyzed for *IL‐6* mRNA expression after 6 h and IL‐6 secretion after 24 h. (S, T) MADD patients derived dermal fibroblasts (P1) were electroporated with dCas9 and JunB guide RNA for CRISPR activation. Collected cells were seeded and the confluent cells were stimulated with LPS 400 ng/mL for 24 h, blotted for JunB and TLR4, and analyzed for IL‐6 secretion. Data in figures (B, G, Q) represent mean ± SEM of four cell culture experiments. Blots (C, E) and (H, M–O) are representatives of at least two independent experiments, while blot in figure (S) was performed once. Data in figure (R and T), represent values of technical ELISA replicates from a single cell culture experiment. *Represents significance compared to healthy controls or untreated control cells (*p < 0.05, **p < 0.01, ***p < 0.001, and ****p < 0.0001, unpaired *t* test).

As the cytokine response of MADD cells with *ETFDH* deficiency to stimulation with LPS was impaired, we speculated that components of the TLR4 signaling pathway could be affected (Figure 2A). Indeed, cells from MADD patients displayed markedly reduced *TLR4* mRNA expression levels when compared to cells from healthy human controls (Figure 2B). Because of the reduced expression of TLR4 we investigated the proximal signaling event of Interleukin‐1 Receptor‐Associated‐Kinase 1 (IRAK1) phosphorylation (pIRAK1). Here, pIRAK1 seemed to be reduced both at basal level as well as in response to LPS (Figure 2C,D). By contrast, pIRAK1 levels were clearly increased in response to stimulation with IL‐1β suggesting that IRAK1 is functional in these cells (Figure 2E,F). Interestingly, the treatment of cells from healthy controls (C2) with siRNA targeting *ETFDH* also resulted in reduced expression of *TLR4* mRNA (Figure 2G  and in reduced pIRAK1 levels (Figure 2H,I).

We then decided to probe the expression of other TLR4‐signaling components besides TLR4 itself and IRAK1. The hetero‐dimeric transcription factor AP1 is crucial for IL‐6 induction and TLR4‐induced transcriptional activation. AP1 functions as a dimer in different combinations with members of the Jun family (c‐Jun, JunB, and JunD) and the Fos family (c‐Fos, FosB, Fra1, and Fra2).^32^ Functional involvement of cAMP response element (CRE)‐binding protein (CREB) has also been found in IL‐6 gene expression. Here, we could observe a notably reduced expression of JunB as well as slightly reduced expression levels of cFOS and CREB in cells from MADD patients whereas c‐Jun was found to be equally expressed between patient and control cells (Figure 2J and Figure S2). We further investigated the expression of JunB in controls as well as in MADD cells 24 h after LPS stimulation. Here, LPS stimulation led to increased expression of JunB in cells from controls (C1, C2), whereas the expression was either below detection levels or failed to increase in response to LPS in MADD cells (P1, P2) (Figure 2K,L). Furthermore, we found that inhibition of *ETFDH* by siRNA or by CRISPR/Cas9 mediated knock‐out was sufficient to also reduce JunB expression levels hereby connecting reduced JunB expression and responsiveness to LPS to the genotype of MADD patients (Figure 2M,N and Figure S3). To test if suppression of JunB was sufficient to suppress IL‐6 expression, we performed siRNA mediated JunB silencing in control cells C2. JunB silencing significantly decreased LPS induced IL‐6 mRNA and IL‐6 secretion. Importantly, JunB silencing also decreased basal level of IL‐6 secretion (Figure 2O–R). This highlighted the crucial role of JunB in IL‐6 response and infers that its reduced expression might be one of the responsible factors for the impaired IL‐6 secretion in patient cells. This inspired us to activate JunB in patient cells to test if this would be sufficient to restore the IL‐6 response. We performed CRISPR activation for JunB in MADD patient cells using specific guide RNA (gRNA) and nuclease‐dead Cas9 (dCas9). We observed a significant increase in JunB expression upon CRISPR activation. However, neither TLR4 expression nor IL‐6 induction was rescued by the forced expression of JunB, suggesting that although JunB is a contributing factor, additional factors are significant for the impaired LPS‐mediated signaling response in MADD (Figure 2S,T).

### 3.3 Deleterious *ACADVL* gene variants also cause impaired responses to LPS

**FIGURE 3:**
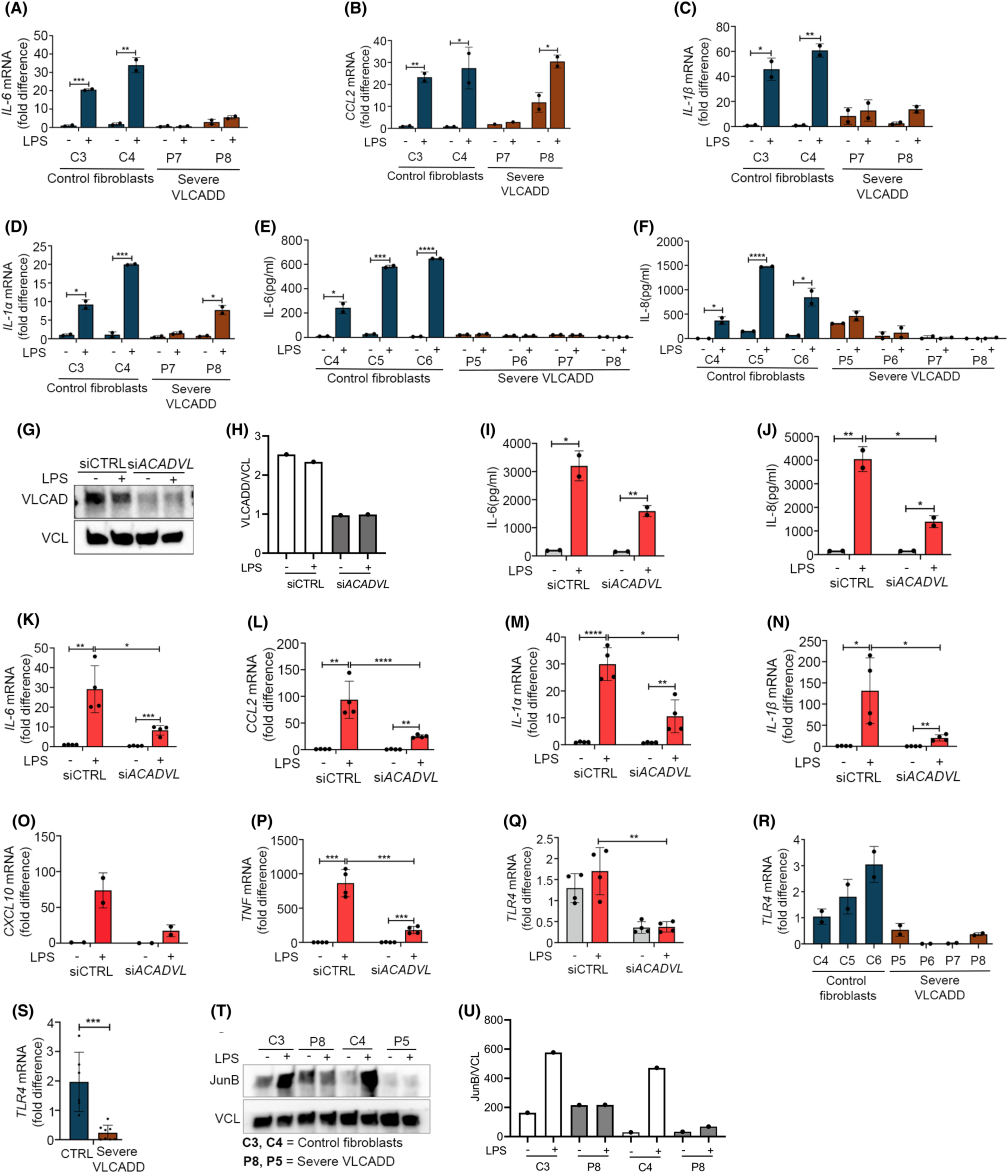
Reduced LPS responsiveness in severe VLCADD patient cells. Primary dermal fibroblasts from healthy controls (C4‐6) and primary dermal fibroblasts derived from severe VLCADD patients (P5‐8) stimulated with LPS 400 ng/mL were analyzed for (A–D) cytokines mRNA (IL‐6, CCL2, IL‐1β, and IL‐1α) expression by RT‐QPCR at 6 h and (E, F) IL‐6, IL‐8 secretion at 24 h. (G, H) Primary dermal fibroblasts from a healthy control (C4) transfected with scrambled siRNA or *ACADVL* siRNA for 72 h were stimulated with LPS 400 ng/mL, blotted and quantified for VLCAD expression. Then the cells were analyzed for (I, J) cytokines secretion (IL‐6, IL‐8) at 24 h, (K‐P) cytokines mRNA (*IL‐6*, *CCL2*, *IL‐1α*, *IL‐1β*, *CXCL10*, *TNF*), and (Q) TLR4 mRNA expression by RT‐QPCR at 6 h. (R, S) Primary dermal fibroblasts from healthy controls (C4‐6) and primary dermal fibroblasts derived from severe VLCADD patients (P5‐8) were analyzed for basal level TLR4 mRNA expression. (T, U) The cells were stimulated with LPS 400 ng/mL for 24 h, blotted and quantified for JunB. Data in figures (A–F) represent mean ± SEM of two cell culture experiments, while data in figures (I–Q) are presented as mean ± SEM of four cell culture experiments. Blots in figures (G and T) were performed twice in independent experiments. *Represents significance compared to healthy controls or untreated control cells (*p < 0.05, **p < 0.01, ***p < 0.001, and ****p < 0.0001, unpaired *t* test).

To investigate if the observed inability to respond appropriately to stimulation with LPS was specific to MADD patients or a common trait in lcFAODs, we investigated fibroblasts from patients with VLCADD. This line of experiments included cells from healthy donors (control cells C3‐6), cells from patients with severe VLCADD (P5‐P8), and with mild VLCADD (P3 and P4) expressing disease‐causing variants of *ACADVL* (Table S1).^33^, ^34^, ^35^ We included patients fibroblasts P7, P8 and compared with control fibroblasts C3, C4 for mRNA study. Interestingly, we observed that similarly to MADD patient cells, LPS stimulation did not induce significant expression of *IL‐6*. In contrast, VLCADD patients cells P8 displayed some induction of *CCL2* and IL‐1α at the mRNA level (Figure 3A–D). We also looked into cytokine secretion with ELISA, where we included patients fibroblasts P6‐8 and compared with control fibroblasts C4‐6. Here also, release of IL‐6 and IL‐8 to the cell culture medium in response to stimulation with LPS was attenuated in cells from severely affected VLCADD patients (Figure 3E,F). As with the MADD deficiencies, we then tried to establish if the deficiencies in *ACADVL* could be linked directly to the insufficient response to LPS. We therefore treated fibroblasts derived from a healthy human control, C4 with siRNA specific for *ACADVL* or with control siRNA (Figure 3G,H). Again, siRNA‐induced deficiency of *ACADVL* led to decreased LPS‐induced release of IL‐6 and IL‐8 to the cell culture medium (Figure 3I,J). This effect was also observed at the mRNA level with reduced induction of *IL‐6*, *CCL2*, *IL‐1α*, *IL‐1β*, *CXCL10*, *and TNFα* (Figure 3K–P). Similar to *ETFDH* silecing, siRNA targeting *ACADVL* resulted in reduced *TLR4* mRNA expression (Figure 3Q). Importantly, as was also observed with cells from the MADD patients, TLR4 expression levels were reduced when comparing VLCADD patient cells with cells from healthy controls (Figure 3R,S). Also, LPS stimulation induced expression of JunB in the control fibroblasts (C3, C4), while in the VLCADD cells (P5, P8), the expression was either not observed or it was not induced (Figure 3T,U).

To further test the linkage between VLCADD and insufficient responses to LPS, we stimulated cells from two patients presenting mild forms of VLCADD and with much higher FAO flux activity. Interestingly, the LPS‐response was less attenuated in these patients with only some repression of IL‐6 responses and clear inductions of IL‐8 (Figure S4A,B). In addition, *TLR4* mRNA expression levels were markedly higher in cells from mild VLCADD patients when compared to cells from patients presenting with severe VLCADD (Figure S4C,D).

Finally, in contrast to the MADD cells, the cytokine response to RNA in the form of poly‐I:C did not seem to be affected in cells from either mild or severe forms of VLCADD (Figure S5). This contrasting responses could be because of differential phenotypes attributed to them. Overall, these data demonstrate a clear link between genes that are important for beta‐oxidation of fatty acids and LPS‐induced inflammatory responses.

### 3.4 Pharmacological blockage of fatty acid oxidation does not impair the IL‐6 response

**FIGURE 4:**
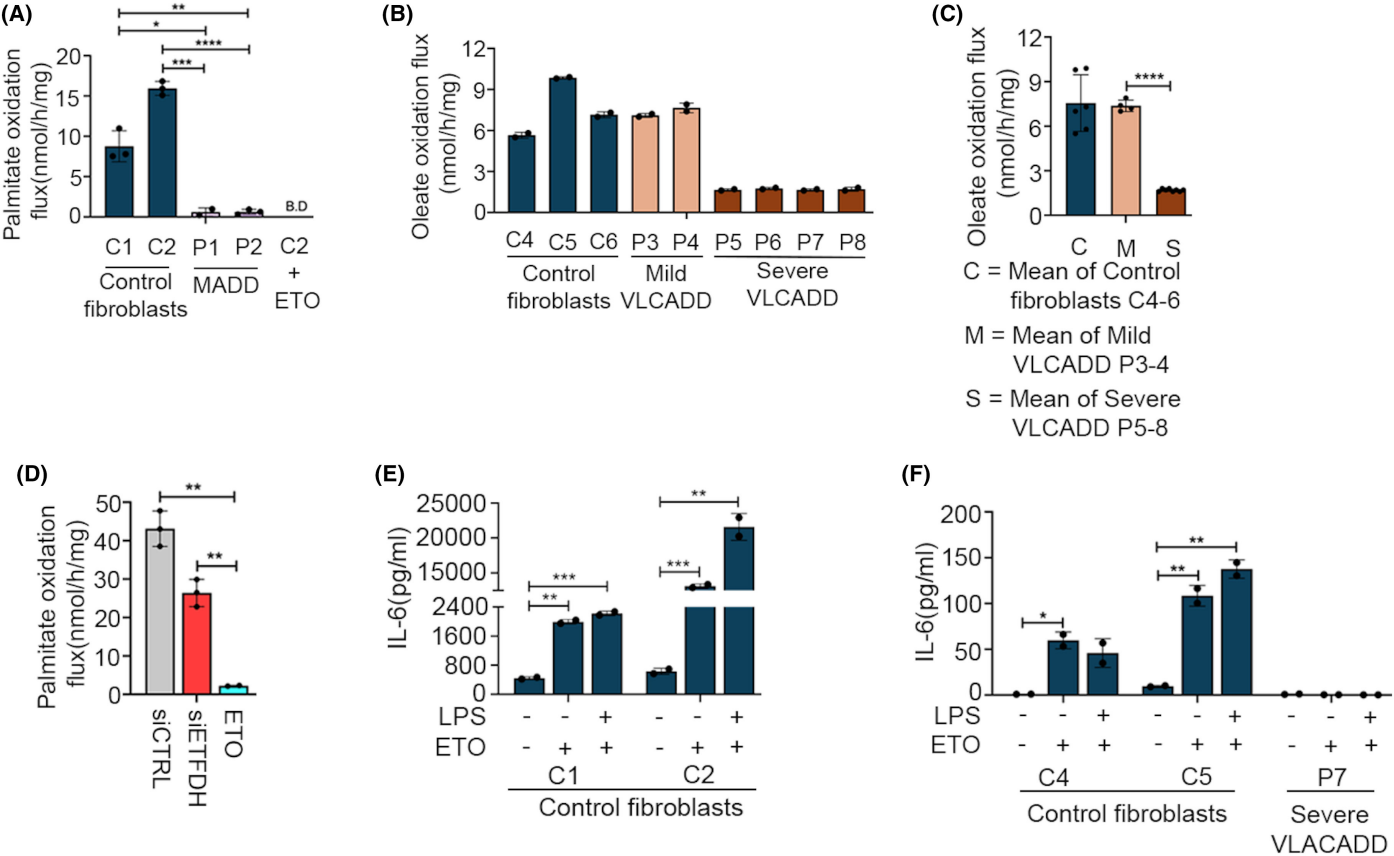
Pharmacological blockage of fatty acid oxidation does not impair cytokine responses to LPS. (A) Palmitate oxidation flux of primary dermal fibroblasts from healthy controls (C1‐2) and dermal fibroblasts derived from two MADD patients (P1‐2). Control fibroblast cells (C2) were preincubated with Etomoxir (ETO) overnight before measurement as a negative control. (B) Oleate oxidation flux of control dermal fibroblasts (C4‐6) and dermal fibroblast cells from severe VLCADD (P5‐8). (C) Group means calculated from data in B, showing overall FAO flux differences in control dermal fibroblasts (C4‐6) and severe VLCADD (P5‐8). (D) Primary dermal fibroblasts from healthy controls C2 were transfected with scrambled siRNA or ETFDH siRNA for 72 h, or preincubated with ETO overnight before palmitate oxidation flux measurement. (E, F) Primary dermal fibroblasts from healthy controls (C1‐2, C4‐5) and severe VLCADD (P7), overnight incubated with ETO were stimulated with LPS 400 ng/mL for further 24 h before measuring IL‐6 by ELISA. Data are presented as mean ± SEM of four cell culture experiments. *p < 0.05, **p < 0.01, ***p < 0.001, and ****p<0.0001; compared to healthy controls or untreated control cells (unpaired *t* test).

Cells from MADD and VLCADD patients have severely compromised lcFAO as demonstrated by reduced lcFAO flux (Figure 4A–C). Inhibition of *ETFDH* by siRNA recapitulated this reduction in lcFAO (Figure 4D). We therefore speculated that inhibition of lcFAO using etomoxir (ETO), which blocks FAO by inhibiting influx of fatty acids from the cytosol into the mitochondria by direct inhibition of CPT1, would affect responsiveness to LPS. Although ETO clearly abrogated lcFAO flux, it did not affect the ability of cells from healthy controls to respond to LPS with secretion of IL‐6, nor did it affect the already reduced IL‐6 expression in patients' cells (Figure 4E,F). These results imply that the impaired response to LPS in cells with genetic deficiencies in *ETFDH* or *ACADVL* might not be due to the blockage of lcFAO per se.

### 3.5 Inhibition of mTOR with rapamycin partially restores basal IL‐6 secretion but not responses to LPS

In order to explore potential drivers of the impaired cytokine response in severe VLCADD and MADD, we performed intracellular metabolomics analyses of dermal fibroblasts from controls, severe VLCADD and MADD, incubated both with and without the addition of LPS, which were subsequently analyzed by UPLC‐mass spectromwtry. We detected 112 unique metabolites, encompassing classes such as amino acids, glycolysis, TCA intermediates, and nucleotides. In dermal fibroblasts from severely affected VLCADD subjects, there was a significant elevation in both essential and non‐essential amino acids. This increase was observed at the basal level, indicating an intrinsic metabolic alteration in the VLCADD cells. Moreover, this trend persisted and became more pronounced following LPS stimulation (Figure S6A,B). The LPS challenge likely exacerbated the metabolic dysregulation already present in these cells, leading to further accumulation of amino acids. Contrary to VLCADD fibroblasts, untreated MADD dermal fibroblasts exhibited an increase in tryptophan and proline only, which was also the most pronouncedly regulated amino acids in the VLCADD fibroblasts. After LPS stimulation, the increase in amino acids disappeared (Figure S6C,D). The reduction in amino acid levels after LPS stimulation could be due to increased metabolic consumption or altered regulatory pathways that are less active in VLCADD cells. Using the metabolic aberrations in patient cells as a starting point, we searched for a number of ways that could be helpful to rescue IL‐6 levels in patient cells. Amino acids are known to influence mTOR signaling by enhancing RAG proteins activation that are essential in recruiting mTOR.^36^ It has been shown that mTOR pathway activation limits NF‐kB activity by enhancing IkBα and thereby suppressing proinflammatory markers in fibroblasts.^37^ Therefore, we wanted to test if mTOR inhibition could be sufficient to fully or partially restore IL‐6 level in patient cells only, where mTOR inhibition is expected. MADD cells (P1) and VLCADD cells (P8) were preincubated with the well‐known inhibitor of mTOR, rapamycin (100 nM) for 24 h, followed by LPS stimulation for a further 24 h without media removal. Rapamycin preincubation decreased phosphorylated mTOR expression and we observed significant increase in basal level IL‐6 secretion in patient cells. However, rapamycin did not restore responsiveness to stimulation with LPS (Figure S6E–G). To further test the involvement of the mTOR pathway, we performed *MTOR* silencing by siRNA in MADD cells (P1, P2). Blotting for phospho‐mTOR showed significantly decreased expressions in si*MTOR* conditions when compared to scrambled siRNA, demonstrating that silencing was effective. Cells transfected with si*MTOR* showed a significantly larger basal level increase in IL‐6 secretion than the respective controls with scrambled siRNA transfection. However, as with rapamycin, responsiveness to LPS was not restored (Figure  S6H–J). Our results imply that mTOR could be involved in repressing IL‐6 expression at the basal level but that inhibition of mTOR is not sufficient to restore LPS sensitivity.

## 4 DISCUSSION

With this work, we provide genetic evidence from human patient samples, that enzymes involved in lcFAO are important for cellular responsiveness to LPS. We observed this phenomenon across six genetically distinct individuals—two MADD patients with distinct *ETFDH* deficiencies and six VLCADD patients with distinct *ACADVL* deficiencies. Our results therefore strongly suggest that a connection between lcFAO enzymes and inflammatory responses exists. This was corroborated by the observation that genetic loss‐of‐function of *ETFDH* and *ACADVL* through siRNA in fibroblasts from healthy subjects generated a similar phenotype.

Dysregulated immune responses with low‐grade inflammation in lcFAOD were recently reported in both animal models as well as patients,^17^, ^21^, ^22^, ^38^ however, the underlying molecular mechanisms remain undiscovered. Further, several studies investigating insulin resistance and obesity have reported that long‐chain saturated fatty acids such as palmitate and long‐chain acylcarnitines that accumulate in lcFAODs can serve as inflammatory triggers. However, the target through which long‐chain acylcarnitines activate an inflammatory response is not yet known.^39^ In this study we learned that blocking lcFAO by etomoxir did in itself not affect responses to LPS indicating that an effect of lcFAODs besides the inability to metabolize fatty acids is likely part of the underlying mechanism. These data therefore also suggest that FAO is not a driver of IL‐6 induction. Dysfunctional inflammatory responses to LPS in leukocytes have recently also been observed in respiratory chain disorders. Karan et al. demonstrate that cytokine responses are blunted in patients with mtDNA deletions encoding respiratory chain proteins, but did not delinate the involved cellular mechanisms.^40^ Patients with respiratory chain disorders may have overlapping clinical, biochemical, and cell biological phenotypes with lcFAODs.^41^, ^42^, ^43^, ^44^

In the present study, we studied primary dermal fibroblasts that are available from diagnostic evaluation of the rare and often deceased lcFAOD patients. Because of the inborn error, the genetic lcFAO defect exist in all cells, implicating that our finding potentially could have broader tissue expression and pathological consequences. Importantly, besides leukocytes and fibroblasts, multiple cell types, including myocytes, adipocytes and neurons, are known to secrete IL‐6, where it can not only cause inflammation but also play a protective role by regulating metabolic function and survival/regeneration of the tissue upon acute stress exposure.^45^ As such, our findings may be key to the understanding of the acute, fulminant onset of metabolic decompensation, in particularly rhabdomyolysis, that is a major burden to the patients, and which often is triggered by infections.^14^, ^15^, ^16^, ^17^, ^18^, ^19^, ^20^ TLR4 is an important member of the toll‐like receptor family and is broadly expressed in human cells and tissue, that is, liver, skin, heart, muscle, and adipocytes.^46^, ^47^, ^48^ Our studies demonstrate that cells derived from patients with lcFAODs, with permanent blockage of lcFAO have constantly decreased TLR4 levels. Further, we link decreased expression of important transcription factors that can interact with the IL‐6 promoter for mRNA synthesis to the impaired IL‐6 response. This could possibly explain the lack of basal IL‐6 response in patients' cells and also provides an opportunity to restore them. In fact, our results indicate that mTOR activation could be involved in repressing IL‐6 expression at the basal level but that inhibition of mTOR is not sufficient to restore LPS sensitivity. This implies that other factors are involved in controlling the blunted IL‐6 responses. Besides NF‐κB, STAT3 is important in regulating LPS‐induced IL‐6 production. Whereas NF‐κB primarily is responsible for initiating IL‐6 transcription in response to LPS, STAT3 acts to modulate the response through feedback mechanisms that can amplify or decrease IL‐6 production, depending on cellular needs and conditions.^49^ This highlights the complex interplay between various signaling pathways in maintaining immune‐inflammatory homeostasis. Future studies may show if some aspects of the cellular pathology in lcFAODs can disrupt these feedback loops, leading to altered TLR4 expression and responsiveness to stimuli like LPS. Together, this study links inborn errors in long‐chain fatty acid oxidation to insufficient responses to LPS. Eventhough, our studies do not identify the exact driver(s) of the defect, it is likely that chronic low‐level inflammasome activation or other persistently activated stress responses in lcFAOD may be driving the molecular changes that affect cells' ability to mount an inflammatory response to an acute immunogenic stimuli. Thus, it would be relevant to test immune‐metabolic homeostasis collapse as a model for acute and reversible metabolic decompensation and rhabdomyolisis in lcFAODs.

## AUTHOR CONTRIBUTIONS

*Conceptualization*: S.M., R.O., K.T., C.H., and R.H. *Methodology*: S.M., K.T., R.O., C.H., R.H., M.W., J.K., B.S., and S.D. *Validation*: S.M., K.T., and B.S. *Formal analysis*: S.M., K.T., B.S. *Investigation*: S.M. and K.T. *Writing*—*original draft*: S.M. and K.T. *Writing*—*review and editing*: S.M., K.T., R.O., C.H., R.H., J.K., and B.S. *Visualization*: S.M., K.T., and B.S. *Supervision*: R.O., C.H., and R.H.

## FUNDING INFORMATION

This research work was supported by: Ascending Investigator grant to CKH from Novo Nordisk Foundation, the Independent Research Fund—Denmark, Fhv. Dir. Leo Nielsen & Hustru Karen Margrethe Nielsens Legat for Lægevidenskabelig Grundforskning, Ester M. & Konrad Kristian Sigurdssons Dyreværnsfond, Beckett‐fonden, Kong Christian IX & Dronning Louises Jubilæumslegat, Christian Larsen & Dommer Ellen Larsens Legat, Direktør Emil C. Hertz & hustru Inger Hertz' fond and A.P. Møller Fonden to CKH. JK was supported by the Netherlands Organization for Scientific Research by receiving a VENI grant from ZonMW (91619098), and was supported by the Dutch Heart Foundation (Senior Scientist Dekker grant (03‐004‐2021‐T045)). SM was supported by an EliteForsk travel grant from Independent Research Fund Denmark (9095‐00046B), PhD Scholarship from Lundbeckfoden (R263‐2017‐4384), Christian og Ottilia Brorsons Rejselegat, Helga og Peter Kornings Fond and Max Nørgaard og Hustru Magda Nørgaards Legat. Work in the Houtkooper lab is financially supported by a Human Measurement models grant from the Netherlands Organization for Scientific Research (NWO) (no. 18953), and work performed at Research Unit for Molecular Medicine is financially supported by the Aarhus County Research Initiative.

## DISCLOSURES

The authors disclose no conflict of interests.

## Supporting information


**Figure S1.** Cytokines and TLR3 responses in MADD.
(A, B) Primary dermal fibroblasts from healthy controls (C1‐2) and primary dermal fibroblasts derived from MADD (P1‐2) were stimulated with LPS 400 ng/mL for 24 h or preincubated overnight with poly I:C 2 μg/mL and analyzed for CXCL10 and IL‐6 secretion by ELISA respectively. (C) Basal level of TLR3 mRNA expression was analyzed in healthy controls (C1‐2) and primary dermal fibroblasts derived from MADD (P1‐2). Data presented as mean ± SEM, and representative of four and two cell culture experiments in A–C respectively. *Represents significance compared to untreated control cells (***p < 0.001, and ****p < 0.0001, unpaired *t* test).

**Figure S2.** IL‐6 promoter linked transcription factors upon LPS stimulation.
Primary dermal fibroblasts from healthy controls (C1‐2) and MADD fibroblasts (P1) stimulated with LPS 400 ng/mL were blotted for IL‐6 promoter linked transcription factors expressions. Blots were performed twice in the independent experiments.
**Figure S3.** ETF‐QO knockout in control fibroblasts.
Primary dermal fibroblasts from healthy controls (C2) were electroporated with ETFDH sgRNA for ETF‐QO knockout and the cells were blotted for ETF‐QO, along with wild type (WT) cells. Knockout was performed as single and double knockout in a single experiment.

**Figure S4.** Mild VLCADD patient cells show LPS responsiveness.
Primary dermal fibroblasts derived from mild VLCADD patients (P3‐4) and severe VLCADD patients (P5‐8) stimulated with LPS 400 ng/mL were analyzed for (A, B) IL‐6, IL‐8 secretion at 24 h and (C) TLR4 mRNA expression by RT‐QPCR at 6 h. (D) Group means calculated from data in C, showing representative TLR4 mRNA expression. Data presented as mean ± SEM of two cell culture experiments. *Represents significance compared to untreated control cells (**p < 0.01, ***p < 0.001, and ****p < 0.0001, unpaired *t* test).
**Figure S5.** Mild and severe VLCADD patient cells show proper responses to polyIC.
Primary dermal fibroblasts from healthy controls (C5‐6) and primary dermal fibroblasts derived from mild VLCADD (P3‐4), and severe VLCADD (P5‐8) patients’ cells were preincubated overnight with poly I:C 2 μg/mL and analyzed for IL‐6 secretion by ELISA. Data presented as mean ± SEM from two cell culture experiments. ****p < 0.0001; compared to untreated control cells (unpaired *t* test).

**Figure S6.** Inhibition of mTOR with rapamycin partially restores basal IL‐6 secretion but not responses to LPS. (A–D) Volcano plot of metabolomics performed using MS/MS. (A, B) Primary dermal fibroblasts derived from healthy controls (*N* = 4, C3‐6) and severe VLCADD (*N* = 4, P5‐8), (A) without and (B) with LPS 400 ng/mL stimulation. (C‐D) Primary dermal fibroblasts derived from healthy controls (*N* = 2, C1‐2) and severe MADD (*N* = 2, P1‐2), (C) without and (D) with LPS 400 ng/mL stimulation. (E–J) MADD (P1‐2) and VLCADD (P8) patient derived dermal fibroblasts were preincubated with 100 nM rapamycin for 24 h or transfected with scrambled siRNA and siMTOR for 72 h and stimulated with LPS 400 ng/mL for 24 h. Cells were then analyzed for IL‐6 secretion and blotted for phospho mTOR. Data in E‐J represent 3 to 4 ELISA technical replicates from a cell culture experiment. *Represents significance compared to untreated control cells (*p < 0.05, **p < 0.01, ***p < 0.001, and ****p < 0.0001; unpaired *t* test).

**Table S1.** Patient cell lines genotypes.

**Table S2.** TaqMan® Gene Expression Assays.

## Data Availability

The data that support the findings of this study are available in the methods and/or supplementary material of this article. In addition, the other relevant data are available by request from the authors.
