# Supplementary figures and images for: Human inborn errors of long‐chain fatty acid oxidation show impaired inflammatory responses to TLR4‐ligand LPS
Source: FASEB Bioadv. 2024 Aug 19;6(9):337–50. doi: 10.1096/fba.2024-00060 (PMC11467727; doi:10.1096/fba.2024-00060)

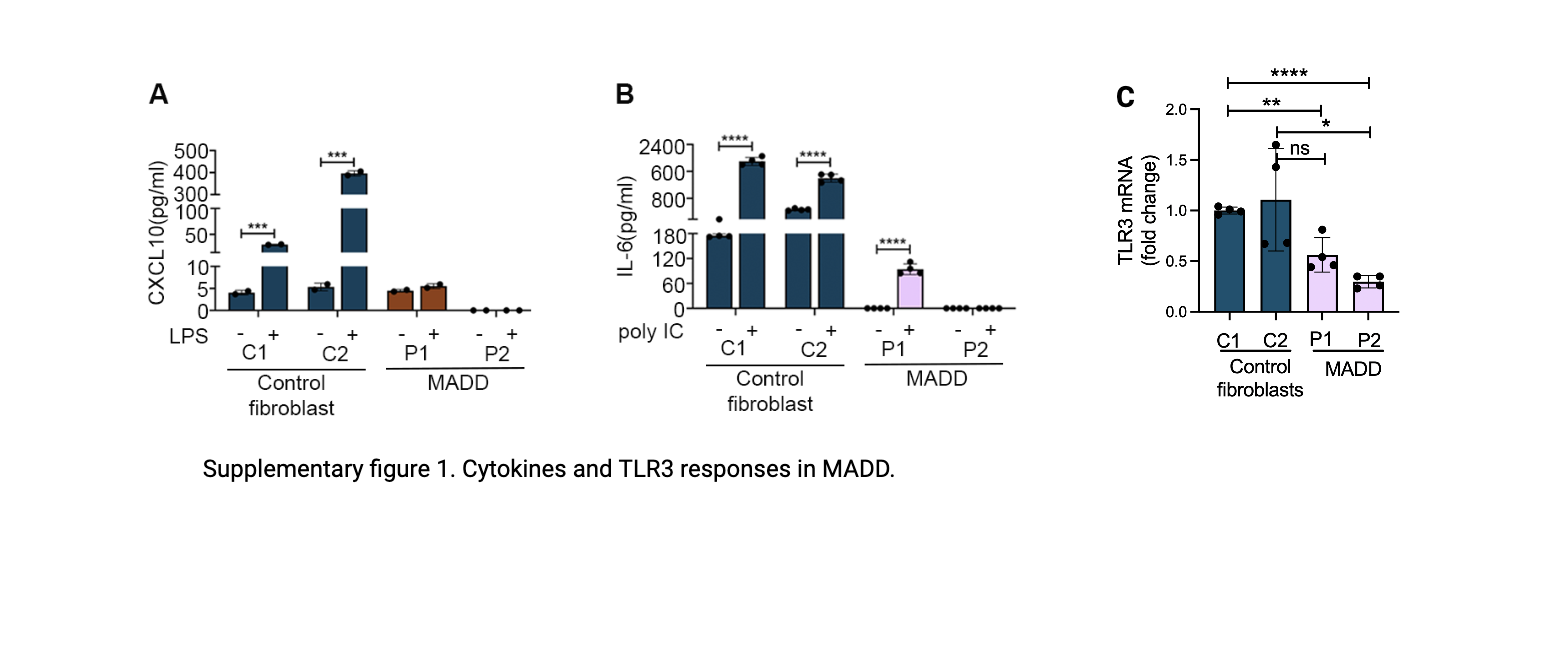

Supplement: Supplementary file 1 — Figure S1. Cytokines and TLR3 responses in MADD. (A, B) Primary dermal fibroblasts from healthy controls (C1‐2) and primary dermal fibroblasts derived from MADD (P1‐2) were stimulated with LPS 400 ng/mL for 24 h or preincubated overnight with poly I:C 2 μg/mL and analyzed for CXCL10 and IL‐6 secretion by ELISA respectively. (C) Basal level of TLR3 mRNA expression was analyzed in healthy controls (C1‐2) and primary dermal fibroblasts derived from MADD (P1‐2). Data presented as mean ± SEM, and representative of four and two cell culture experiments in A–C respectively. *Represents significance compared to untreated control cells (***p < 0.001, and ****p < 0.0001, unpaired t test). [file FBA2-6-337-s004.png]

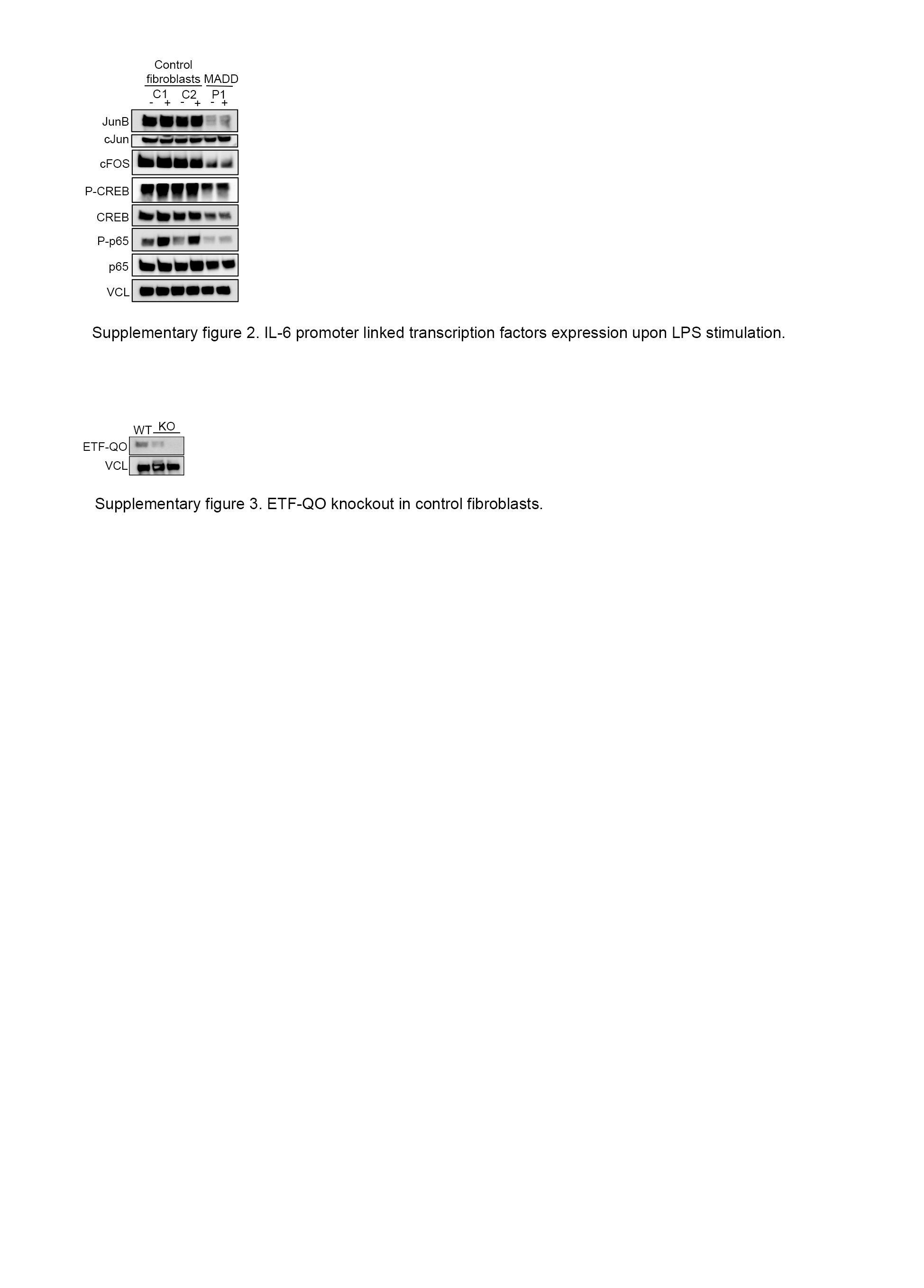

Supplement: Supplementary file 2 — Figure S2. IL‐6 promoter linked transcription factors upon LPS stimulation. Primary dermal fibroblasts from healthy controls (C1‐2) and MADD fibroblasts (P1) stimulated with LPS 400 ng/mL were blotted for IL‐6 promoter linked transcription factors expressions. Blots were performed twice in the independent experiments. Figure S3. ETF‐QO knockout in control fibroblasts. Primary dermal fibroblasts from healthy controls (C2) were electroporated with ETFDH sgRNA for ETF‐QO knockout and the cells were blotted for ETF‐QO, along with wild type (WT) cells. Knockout was performed as single and double knockout in a single experiment. [file FBA2-6-337-s005.png]

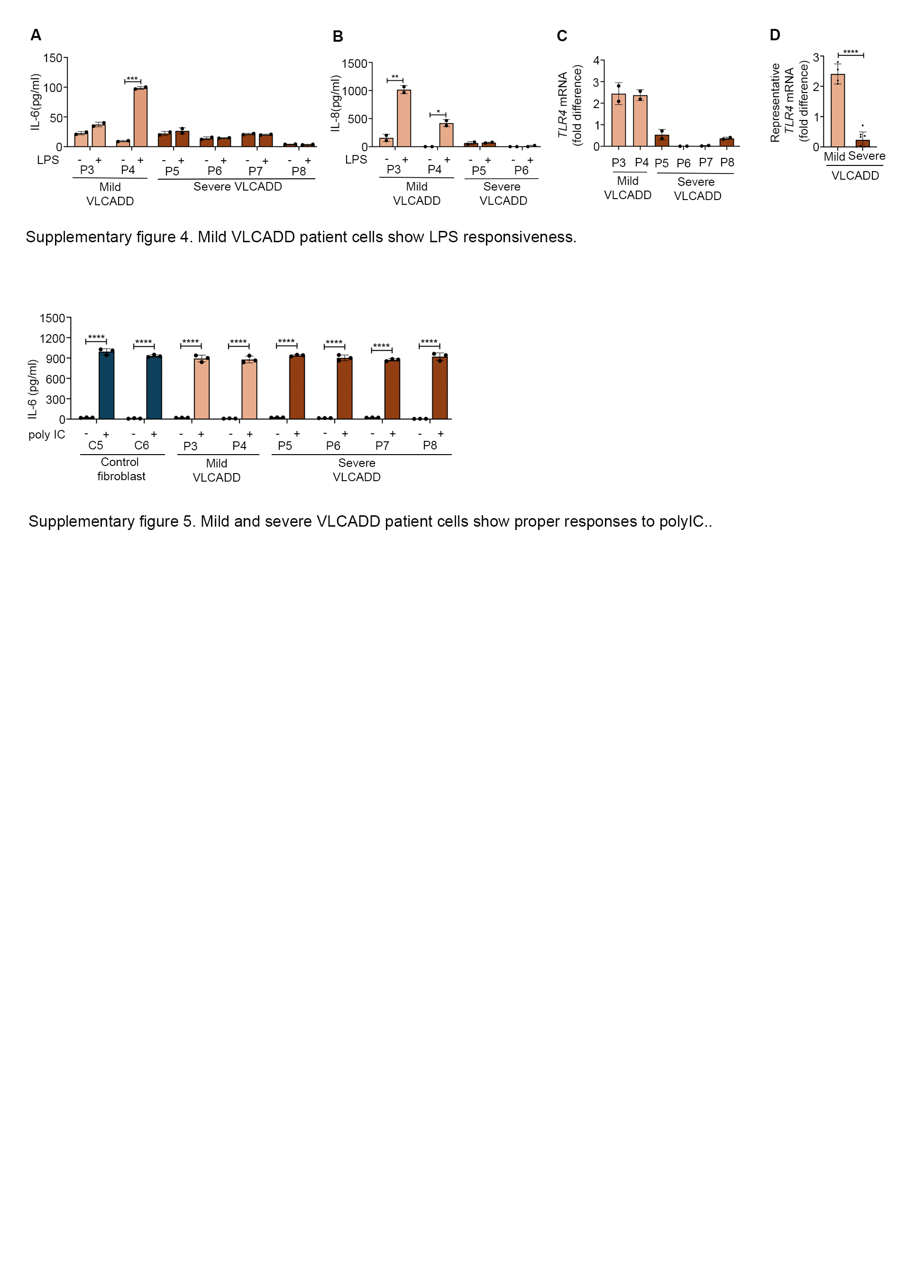

Supplement: Supplementary file 3 — Figure S4. Mild VLCADD patient cells show LPS responsiveness. Primary dermal fibroblasts derived from mild VLCADD patients (P3‐4) and severe VLCADD patients (P5‐8) stimulated with LPS 400 ng/mL were analyzed for (A, B) IL‐6, IL‐8 secretion at 24 h and (C) TLR4 mRNA expression by RT‐QPCR at 6 h. (D) Group means calculated from data in C, showing representative TLR4 mRNA expression. Data presented as mean ± SEM of two cell culture experiments. *Represents significance compared to untreated control cells (**p < 0.01, ***p < 0.001, and ****p < 0.0001, unpaired t test). Figure S5. Mild and severe VLCADD patient cells show proper responses to polyIC. Primary dermal fibroblasts from healthy controls (C5‐6) and primary dermal fibroblasts derived from mild VLCADD (P3‐4), and severe VLCADD (P5‐8) patients’ cells were preincubated overnight with poly I:C 2 μg/mL and analyzed for IL‐6 secretion by ELISA. Data presented as mean ± SEM from two cell culture experiments. ****p < 0.0001; compared to untreated control cells (unpaired t test). [file FBA2-6-337-s002.png]

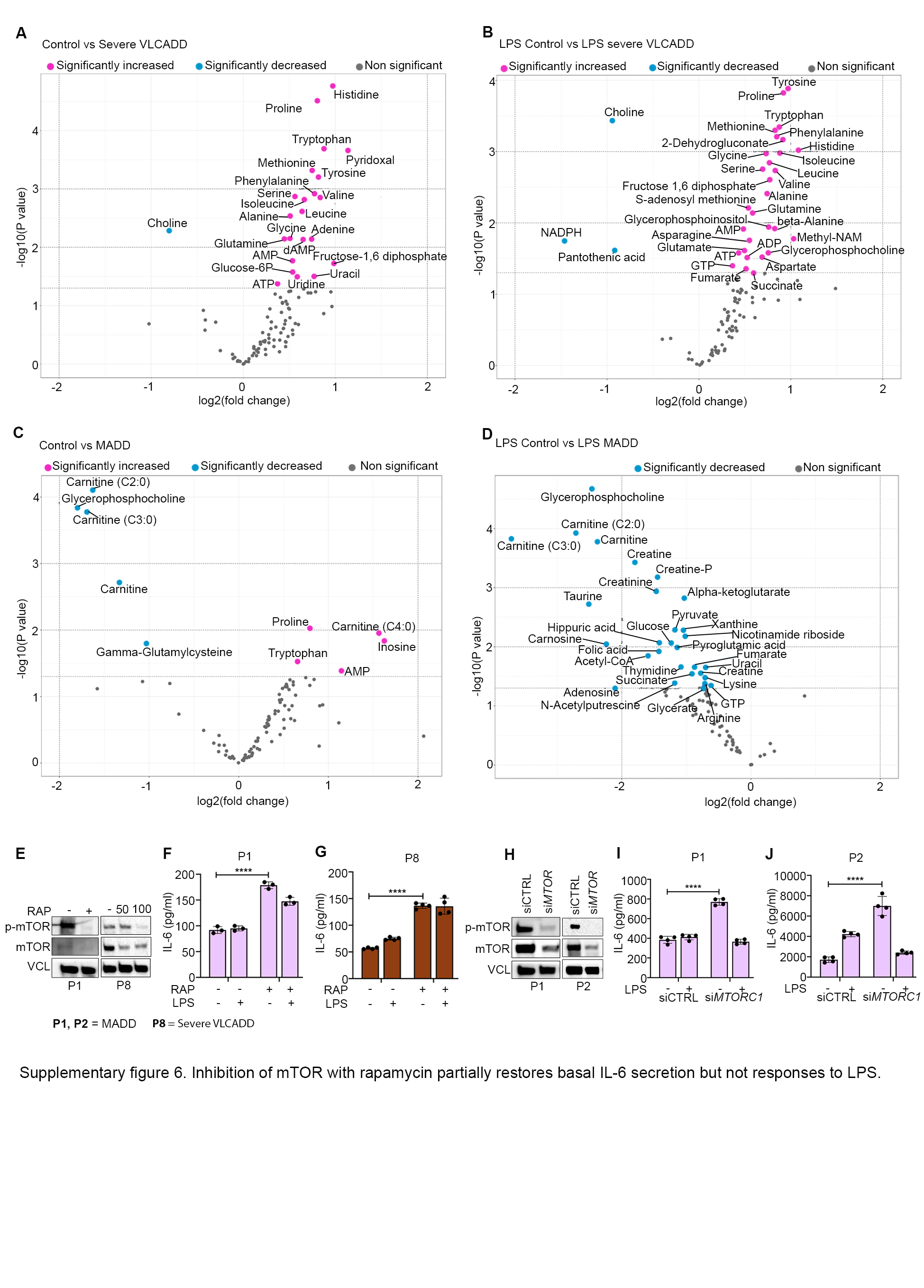

Supplement: Supplementary file 4 — Figure S6. Inhibition of mTOR with rapamycin partially restores basal IL‐6 secretion but not responses to LPS. (A–D) Volcano plot of metabolomics performed using MS/MS. (A, B) Primary dermal fibroblasts derived from healthy controls (N = 4, C3‐6) and severe VLCADD (N = 4, P5‐8), (A) without and (B) with LPS 400 ng/mL stimulation. (C‐D) Primary dermal fibroblasts derived from healthy controls (N = 2, C1‐2) and severe MADD (N = 2, P1‐2), (C) without and (D) with LPS 400 ng/mL stimulation. (E–J) MADD (P1‐2) and VLCADD (P8) patient derived dermal fibroblasts were preincubated with 100 nM rapamycin for 24 h or transfected with scrambled siRNA and siMTOR for 72 h and stimulated with LPS 400 ng/mL for 24 h. Cells were then analyzed for IL‐6 secretion and blotted for phospho mTOR. Data in E‐J represent 3 to 4 ELISA technical replicates from a cell culture experiment. *Represents significance compared to untreated control cells (*p < 0.05, **p < 0.01, ***p < 0.001, and ****p < 0.0001; unpaired t test). [file FBA2-6-337-s003.png]
