# Supplementary material for: Human inborn errors of long‐chain fatty acid oxidation show impaired inflammatory responses to TLR4‐ligand LPS
Source: FASEB Bioadv. 2024 Aug 19;6(9):337–50. doi: 10.1096/fba.2024-00060 (PMC11467727; doi:10.1096/fba.2024-00060)
Supplement: Supplementary file 5 — Table S1. Patient cell lines genotypes. [file FBA2-6-337-s006.docx]

**Supplementary table 1.** Patient cell lines genotypes

| **MADD**  **ID** | **Nucleotide change**  **(*ETFDH*)** | **Protein change**  **(ETFQO)** | **Literature** | **Severity^*^** |
| --- | --- | --- | --- | --- |
| P1 | c.[1234G>T];[1852T>C] | p.[Glu412Ter];[Ter618Gln] | NA | Severe |
| P2 | c.[158A>G];[158A>G] | *Missplicing and protein loss* | Olsen *et al.* 2014 | Severe |
| **VLCADD**  **ID** | **Nucleotide change**  **(*ACADVL*)** | **Protein change**  **(VLCAD)** | **Literature** | **Severity** |
| P3 | c.[541dupC];[1072A>G] | p.[Val283Ala];[Glu381del] | Diekman *et al.* 2015 | Mild |
| P4 | c.[848T>C];  [1444_1448delAAGGA] | p.[Pro35LeufsX26];  [Val283Ala] | Andresen *et al*. 1996; Diekman *et al.* 2015 | Mild |
| P5 | c.[798delAGTT];  [798delAGTT] | p.[Val267Glnfs*8];  [Val267Glnfs*8] | Chegary *et al.* 2009 | Severe |
| P6 | c.[104delC];[104delC] | p.[Pro35LeufsX26];  [Pro35LeufsX26] | Diekman *et al.* 2015 | Severe |
| P7 | c.[1322G>A];[1322G>A] | p.[Gly441Asp];[Gly441Asp] | Diekman *et al.* 2015 | Severe |
| P8 | c.[848T>C];  [1444_1448delAAGGA] | p.[Pro35LeufsX26];  [Val283Ala] | Andresen *et al*. 1996; Diekman *et al.* 2015 | Severe |
| Abbreviations; NA, not applicable.  * Severity score is based upon a combination of genotype, fatty acid flux and/or clinical presentation. Severe and mild is referenced to the classification reported by Diekman *et al.* 2015, Bleeker *et al.* 2019, Olsen *et al.* 2003. | | | | |
