# Supplementary material for: Human inborn errors of long‐chain fatty acid oxidation show impaired inflammatory responses to TLR4‐ligand LPS
Source: FASEB Bioadv. 2024 Aug 19;6(9):337–50. doi: 10.1096/fba.2024-00060 (PMC11467727; doi:10.1096/fba.2024-00060)
Supplement: Supplementary file 6 — Table S2. TaqMan® Gene Expression Assays. [file FBA2-6-337-s001.docx]

**Supplementary table 2.** TaqMan® Gene Expression Assays

| **Supplementary table 2. TaqMan® Gene Expression Assays** | |
| --- | --- |
| **Gene** | **Gene Assay ID** |
| *IL6* | Hs00174131_m1 |
| *CCL2* | Hs00234140_m1 |
| *IL-1β* | Hs01555410_m1 |
| *hTNF-α* | Hs00230464_m1 |
| *IL-1α* | Hs00899844_m1 |
| *CXCL10*  *TLR4* | Hs00171042_m1  Hs00152939_m1 |
